# The physiology of thoracic duct pressure and flow: A review of the literature

**DOI:** 10.14814/phy2.70742

**Published:** 2026-01-22

**Authors:** Sara Moazzam, Lomani A. O'Hagan, Alys R. Clarke, Peter S. Russell, Anthony R. J. Phillips, John A. Windsor, S. Ali Mirjalili

**Affiliations:** ^1^ School of Medicine The University of Auckland Auckland New Zealand; ^2^ Department of Anatomy and Medical Imaging The University of Auckland Auckland New Zealand; ^3^ Auckland Bioengineering Institute The University of Auckland Auckland New Zealand; ^4^ Department of Surgery, Faculty of Medical and Health Sciences, Surgical and Translational Research Centre University of Auckland Auckland New Zealand; ^5^ Applied Surgery and Metabolism Laboratory, School of Biological Sciences The University of Auckland Auckland New Zealand; ^6^ Faculty of Medical and Health Sciences, Surgical and Translational Research Centre The University of Auckland Auckland New Zealand

**Keywords:** circulation, flow, lymphatic system, physiology, pressure, respiration, systematic review, thoracic duct

## Abstract

The thoracic duct (TD) is the largest vessel of the lymphatic system, transporting interstitial fluid, macromolecules, and immune cells into the venous circulation via the lymphovenous junction. Respiratory and circulatory forces have been proposed as key drivers of TD lymph propulsion; however, the literature reports inconsistent findings. This study systematically reviews the effects of respiration and circulation on TD lymph flow and pressure in humans and non‐human mammals. A systematic review was conducted in accordance with PRISMA guidelines using MEDLINE, Embase, and Google Scholar databases. Studies published up to August 2025 were included with no language or past date restrictions. Twenty‐three human and animal studies met the inclusion criteria. Respiratory activity influenced TD flow and/or pressure in 5/6 human and 12/17 animal studies. Circulatory influences were reported in 3/6 human and 8/17 animal studies. Intrinsic TD contractility was described in 3/6 human and 6/17 animal studies and was identified as an independent contributor to lymph propulsion. Overall, reported effects ranged from absent to highly synchronous physiological coupling. Evidence regarding respiratory and circulatory influences on TD lymphodynamics remains inconsistent, reflecting methodological heterogeneity. Findings should be considered hypothesis‐generating and highlight the need for modern imaging and standardized physiological protocols.

## 1 INTRODUCTION

Functioning as a key component of the circulatory system, the lymphatic system is a network of distensible vessels that collect and transport lymphatic fluid back into the central circulation (Dumont, 1975). Propulsion of lymph through this system is achieved by both the intrinsic rhythmic contractility of smooth muscle in lymphatic vessels and by extrinsic forces acting on these vessels from surrounding tissues or organs. Lymph formed in the interstitial space drains into initial lymphatic capillaries, progresses through collecting lymphatic vessels, converges into regional lymph nodes and larger lymphatic trunks, and ultimately enters the cysterna chyli before ascending via the thoracic duct (TD) towards the lymphovenous junction (Solari et al., 2020). Intraluminal valves exist within lymphatic vessels to prevent the backflow of lymph (Breslin, 2014). The human body generates approximately 8 liters of lymphatic fluid per day, which contains proteins, lipids, metabolites, electrolytes, vitamins, immunoglobulins, and immune cells (Levick & Michel, 2010; Morris & Courtice, 1977; Zilversmit, 1965).

The TD is the largest (and terminal) vessel of the lymphatic system, responsible for lymphatic drainage from the entire body except for the right sides of the head and neck, thorax, and right upper limb, which drain via the right lymphatic duct (Ilahi et al., 2020). The TD originates from the caudal aspect of the cisterna chyli, ascends into the posterior mediastinum via the aortic hiatus, and traverses the thorax between the vertebral column and esophagus. As it ascends, it passes posterior to the aortic arch, extends a few centimeters above the clavicle, and returns to the thorax to empty into the venous circulation—typically at the confluence of the left internal jugular and left subclavian veins, known as the left venous angle (*Gray's anatomy*: *The anatomical basis of clinical practice*, 2021).

Several studies have investigated the relationship between respiration and circulation (especially transmitted arterial pulsations) on TD flow and pressure, in both humans and non‐human mammals (dogs and sheep). Additionally, numerous experiments in humans and animals have demonstrated intrinsic TD contractions in‐vivo, which aid in promoting lymph drainage into the venous system at the lymphovenous junction (LVJ) (Conforti et al., 1955; Hall et al., 1965; Hatta, 1952; Kelly et al., 2022; Kinmonth & Taylor, 1956; Kinnaert, 1973; Onizuka et al., 1997). The intrinsic contractility of the human TD can be both spontaneous and provoked with agonists such as norepinephrine (ex‐vivo) (Telinius et al., 2010). The literature remains divided on the relative contributions of these factors, and the subject has not been thoroughly reviewed in the era of modern medical imaging techniques. The effect of respiration and circulation on the dynamics of lymph movement through the TD has clinical relevance, being implicated in the pathogenesis and pathophysiology of many diseases, including hypertension and heart failure. The failure of TD flow can result in both peripheral and visceral oedema (Balasubbramanian & Mitchell, 2022; Hagan, 2021). This is also relevant to individuals with hypo‐ or hyperventilation, smokers, patients with cardiopulmonary diseases, and those undergoing thoracic surgery (Riemenschneider & Shields, 1981).

Despite longstanding assumptions about respiratory or circulatory drivers of flow, the literature remains strikingly inconsistent, largely due to methodological diversity, small sample sizes, a combination of pediatric and adult subjects, and limited ability to directly visualize real‐time lymph movement. This review therefore aims to map existing evidence and identify knowledge gaps rather than draw firm physiological conclusions.

## 2 MATERIALS AND METHODS

### 2.1 Study protocol

A systematic review of the literature regarding the impact of respiration and circulation on the flow and pressure of TD lymph was undertaken. This review followed the Preferred Reporting Items for Systematic Reviews and Meta‐Analyses (PRISMA) guidelines in its search and selection processes; the completed PRISMA 2020 checklist is provided as File S1. However, given the heterogeneity and limited quality of available studies, the approach functions more accurately as a perspective on the knowledge gap rather than a classical systematic review (LA et al., 2009). Prior to database searching, the study was registered with the Prospective Register of Systematic Reviews (PROSPERO) (registration number: CRD420251085382).

### 2.2 Literature search

The MEDLINE, Embase, Embase Classic and Google Scholar databases were searched using the following search terms: “thoracic duct” OR “lymphovenous junction” AND the combined results of “respiration” OR “circulation” OR “flow” OR “physiology” OR “pressure”. All studies up to August 2025 were included with no language or past date restrictions. Non‐English papers were translated using Google Translate. Forward and backward citation tracking of the primary records was performed along with Google Scholar searches (first 100 results) to identify any relevant literature not found in the primary databases. Historical papers were obtained through the University of Auckland's interlibrary loan service.

### 2.3 Inclusion and exclusion criteria

Duplicate studies were removed, and all articles were title and abstract‐screened against the inclusion/exclusion criteria. Inclusion criteria were human and non‐human mammalian studies, including living, cadaveric, radiological or intra‐operative studies, of either healthy or diseased states. Both in‐vivo and ex‐vivo studies were included. Non‐mammalian studies were excluded. Studies that did not record either respiration or circulation were excluded. Studies that did not subjectively or objectively describe flow or pressure of TD lymph were excluded. Studies were also excluded if the study subject and method were unclear due to language translational difficulties. Potentially relevant studies underwent full‐text review to assess eligibility. Two authors (SM and LO) independently performed the literature searches, title and abstract screening, and full‐text reviews. Discrepancies were compared, and a final group of studies for inclusion was reached through consensus.

### 2.4 Data extraction

Data extraction focused on capturing methodological details, ventilatory state (spontaneous vs. mechanical), subject characteristics, and whether flow or pressure demonstrated synchrony with respiratory or cardiac cycles. Due to incompatible units, inconsistent endpoints, and lack of standardized definitions across studies, quantitative synthesis was not feasible. For each eligible study, quantitative and qualitative data regarding TD lymph flow and/or pressure were extracted by a single author (SM) and checked by a second (LO).

## 3 RESULTS

After removing duplicated studies, a total of 3249 articles were screened, including 41 that were obtained through manual searches. Of these, 40 were identified as appropriate for full‐text review.

**FIGURE 1:**
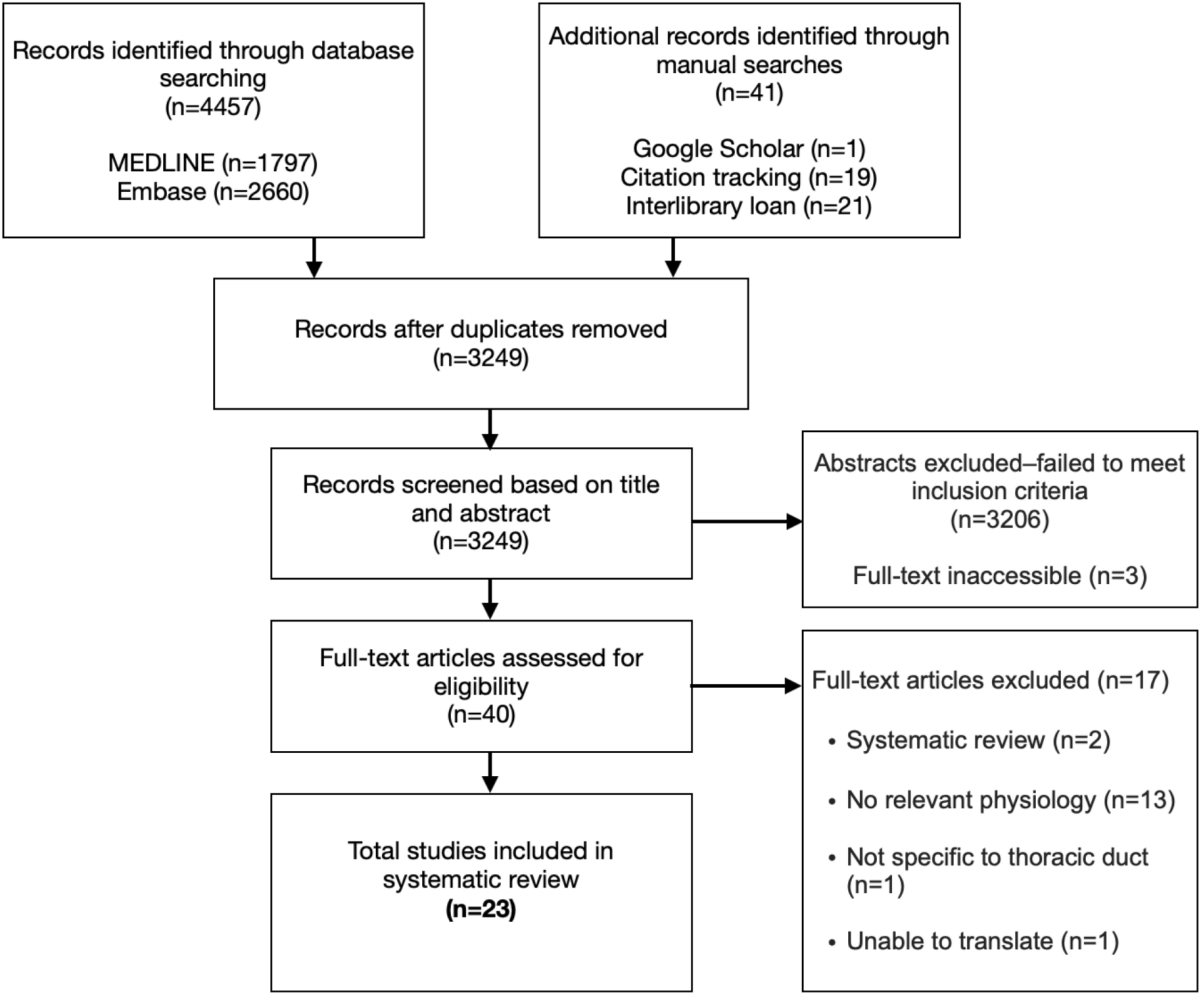
Flow chart of literature search strategy based on PRISMA guidelines.

In total, 23 articles met the inclusion criteria and were included in this review (Figure 1). This represents fewer than 1% of screened studies, highlighting not only the scarcity of quality research in this field but also the substantial inconsistency in methodology that limits interpretation.

### 3.1 Impact of respiration and circulation on flow and pressure in humans

**FIGURE 2:**
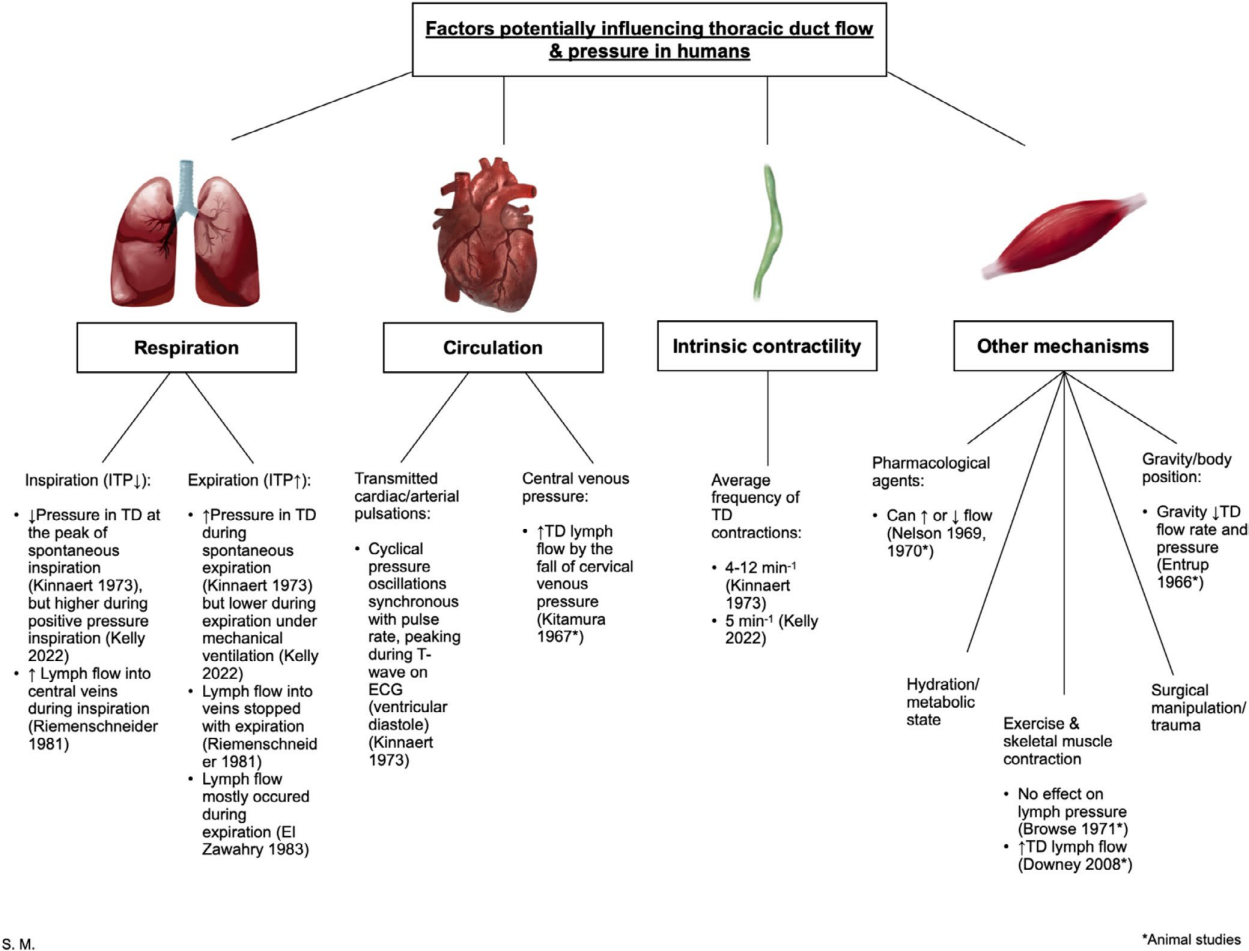
Summary of the factors potentially influencing TD flow and pressure in humans (Browse et al., 1971; Downey et al., 2008; El Zawahry et al., 1983; Entrup et al., 1966; Kelly et al., 2022; Kinnaert, 1973; Kitamura, 1967; Nelson et al., 1969, 1970; Riemenschneider & Shields, 1981).

In‐vivo human studies investigating TD physiology are limited in number and heterogeneous in methodology, though they broadly fall into three categories: radiographic assessments, direct cannulation of the duct, and pressure recordings during surgical or interventional procedures. Although several studies attempted to characterize the mechanisms of lymph flow, no clear consensus has emerged regarding the relative contributions of respiratory movements, cardiac pulsations, and intrinsic contractility (Figure 2). Many studies involved small sample sizes or unspecified participant numbers and employed differing protocols to assess TD flow and pressure.

Intraoperative findings and radiographic investigations offer early insights into the extrinsic drivers of lymph propulsion. Kinmonth and Taylor (1956) first observed spontaneous, rhythmic contractions every 10–15 s during a surgical procedure (Kinmonth & Taylor, 1956), but subsequent lymphangiographic series have failed to reproduce these findings consistently. For example, one study of 25 patients with and without thoracic disease demonstrated that TD emptying (flow) appeared to be influenced by respiratory maneuvers, particularly coughing, rather than intrinsic contractions, with flow into the venous system occurring most frequently after such efforts (Pomerantz, 1963). Another study of lymphangiograms for tumor staging reported that intrathoracic pressure changes during inspiration were the primary drivers of lymph flow across the LVJ, with intravenous flow ceasing on expiration and lymph flowing cephalad between TD valves, possibly aided by the TD's muscle tone and relatively positive intrathoracic pressure during this phase. Lymph flow appeared to be in volume increments proportional to tidal volume. Aortic pulsations were deemed insufficient to drive lymph flow (although it was not explained how this conclusion was reached), and no intrinsic contractions were observed (Riemenschneider & Shields, 1981). These findings factually reported a dominant role for respiratory mechanics, though methodological limitations preclude any definitive conclusions.

Further attempts to delineate the role of intrinsic contractility include a direct TD cannulation study of conscious humans (study size unspecified) under local anesthesia, in which flow appeared to occur primarily during expiration and ceased with voluntary apnea. Minimal transmitted pulsations from the heart or other vessels were observed. The authors proposed that the lower TD was actively contractile, while the upper portion was passively driven by respiratory forces. However, this conclusion was largely inferred from microscopic anatomical observations, as no direct evidence of contractile activity was presented, nor were pressure or flow rates quantified (El Zawahry et al., 1983).

Studies measuring TD pressure intraoperatively offer more direct physiological data. In one such study, patients with chronic kidney disease undergoing surgical TD drainage demonstrated large oscillations in TD pressure, with peak values recorded during expiration (2.6 to 22 mmHg) and nadirs seen at the peak of inspiration (−5 to 17 mmHg). Smaller, cardiac‐synchronous oscillations (~4 mmHg) were observed to peak during ventricular diastole. In one quarter of the patients, pressure waves were independent of respiration or heart rate, yet no TD contractions were visualized intraoperatively (Kinnaert, 1973).

A more recent retrospective study analyzed TD pressure tracings during lymphatic interventional procedures in 25 pediatric patients with congenital heart disease and lymphatic complications, all of whom had undergone Fontan surgery and were having positive pressure mechanical ventilation (Kelly et al., 2022). Among them, 10 patients underwent a 40–50 s ventilatory pause, allowing assessment of intrinsic TD activity. Intrinsic TD contractions were observed and produced a mean pressure rise of 4 ± 4 mmHg, at a frequency of 5/min, consistent with historical observations (Kinmonth & Taylor, 1956). Notably, TD contractions persisted in all 10 patients during a ventilatory pause but were only observed in six patients during resumed ventilation. Positive pressure ventilation significantly elevated both TD and venous pressures, correlating with the peak inspiratory pressures (Kelly et al., 2022).

Taken together, these human studies (Table 1) underscore the predominant influence of external mechanisms on TD flow and pressure, with supportive but inconsistent evidence for relative contributions of both respiration and circulation. Variation in study techniques, populations, and measurement endpoints significantly limits synthesis.

**TABLE 1:** The summary of impact of respiration and circulation on TD flow and pressure in human studies as reported in the literature (El Zawahry et al., 1983; Kelly et al., 2022; Kinmonth & Taylor, 1956; Kinnaert, 1973; Pomerantz, 1963; Riemenschneider & Shields, 1981).

Author	Subject	Sample size	Method	Flow or pressure synchronous with respiration	Flow or pressure synchronous with heart rate	Intrinsic contractions of TD observed
Kinmonth & Taylor (1956)	Human patient	1	Intraoperative observation	N/A	N/A	Yes
Pomerantz (1963)	Human patients with thoracic disease (including benign and malignant neoplasms, and infectious processes), and healthy human subjects without thoracic disease	25	Fluoroscopic studies where movement of contrast medium in the TD was recorded during normal respiration, exaggerated respiratory movements, coughing, and Valsalva maneuver	Yes, but unspecified if flow synchronous with inspiration or expiration	N/A	No
Kinnaert (1973)	Male patients between 16 and 49 years old with end stage renal failure, undergoing a surgery to drain TD lymph. Anesthetized and spontaneously breathing	8	Intraoperative continuous monitoring of TD pressure using a venous strain‐gauge transducer	Yes, pressure in TD highest during expiration, and lowest at the peak of inspiration	Cyclical oscillations synchronous with heart rate seen, peaking synchronously with T‐wave on ECG	Yes, in 2 out of 8 patients: independent pressure waves observed every 5–15 s
Riemenschneider & Shields (1981)	Human patient(s) undergoing tumor staging	Unspecified	Fluoroscopic lymphangiography, observed with normal respiration and during Valsalva maneuver. No measurements recorded	Yes, lymph was propelled into central veins during inspiration, flow of TD lymph into veins stopped with onset of expiration, and flow of TD lymph stopped entirely with Valsalva	No	No
El Zawahry et al. (1983)	Human patient(s), details unspecified	Unspecified	Cannulation of the cervical portion of the TD under local anesthesia, flow of lymph visually observed	Lymph was observed to mostly flow out during expiration. Voluntarily stopping breathing stopped this flow	Flow pattern seen which was theorized to be related to pulse rate, but no method of confirmation	Study concluded that lower 2/3rds of duct had autonomous activity, but this was not actually observed
Kelly et al. (2022)	Pediatric patients with severe congenital heart defects, during procedures performed under GA and PPV	35	Retrospective study of data from interventional lymphatic procedures. TD pressure and central venous pressure measured in 35 patients during mechanical ventilation, 10 of which were also measured during a ventilatory pause	TD pressure was 16 ± 5 mmHg during expiration, and 18 ± 5 mmHg during positive pressure inspiration	Yes–average contribution to TD pressure from each cardiac pulsation of 0.7 mmHg (±0.5)	TD contractions always present during ventilatory pause and sometimes present during ventilation. Average contraction frequency of 5/min

Abbreviations: GA, general anesthesia; LA, local anesthesia; PPV, positive pressure ventilation; TD, thoracic duct.

### 3.2 Impact of respiration and circulation on TD flow and pressure in non‐human mammals

Compared to human studies, investigations of TD physiology in other mammals are more extensive, although findings lack consensus. These studies have typically involved radiographic imaging, direct cannulation with flow and/or pressure measurement, and comparisons between ventilated and spontaneously breathing models. Wide variation in protocols and outcome measures impedes the synthesis of findings across studies.

Radiographic investigations from the 1960s and 1970s report variable influences of respiration and cardiac activity. A study in 1964 observed rhythmic expulsion of contrast‐enhanced lymph into the venous system, potentially due to mechanical compression of the TD by the left atrium, with faster flow seen proximal to this segment. Manual abdominal compression also increased flow, but normal respiration did not affect the movement of lymph, nor were definite TD contractions visualized (Nusbaum et al., 1964). Other studies failed to find a correlation between cardiac pulsation and lymph flow (Nelson et al., 1969), while some observed rhythmic lymph propulsion even immediately after death, implying intrinsic TD contractility (Hatta, 1952). The latter study also noted flow synchronized with diaphragmatic contractions and ceased when the diaphragm was inactive, suggesting a role for diaphragmatic movement in modulating flow. Physical observations of this phenomenon were not reported.

Direct TD cannulation studies in dogs and sheep have explored similar mechanics. Kitamura et al. observed augmented TD lymph flow with diaphragmatic contraction and reduction when the diaphragm was paralyzed or relaxed (Kitamura, 1967). Conversely, Fazzini et al. found no significant change in flow after bilateral division of phrenic nerves in dogs (Fazzini et al., 1964a). The methodological differences and limited descriptions of flow quantification make it difficult to reconcile these findings. The technique and measurement sensitivity are other likely outcome modulating factors.

Studies in mechanically ventilated non‐human mammals further complicate interpretation of TD physiology. Pilon et al. observed that initiating positive end‐expiratory pressure (PEEP) ventilation in dogs significantly and immediately reduced TD flow (from 0.55 ± 0.15 mL/min to 0.17 ± 0.17 mL/min, *p* < 0.005). This was attributed to increased central venous pressure and intrathoracic compression of lymphatic vessels. Flow gradually returned after 30 minutes, possibly due to increased lymph accumulation from systemic venous hypertension (Pilon & Bittar, 1973). Similarly, Browse et al. reported that lymph flow was faster during inspiration and greater in ventilated than in spontaneously breathing anesthetized dogs. During apnea, the lymph flow was replaced by movement in a bidirectional oscillatory pattern, attributed to transmitted aortic pulsations. In conscious dogs, flow was higher overall and again favored inspiration, with transient cessation observed during breath‐holding (e.g. while the animal was drinking water). No intrinsic contractile activity was described (Browse et al., 1974).

Flowmetry studies using ultrasound transit‐time flowmeters in sheep yielded contrasting results. Onizuka et al. found regular, pulsatile TD lymph flow without clear linkage to respiratory or cardiac cycles, suggesting intrinsic mechanisms. Stopping PEEP ventilation also produced no change in lymph flow pattern (Onizuka et al., 1997). However, Iizuka et al. identified flow wave components consistent with respiration (24 min^−1^), cardiac pulsation (180 min^−1^) and possible intrinsic activity (~0.1 Hz), implying multifactorial regulation (Iizuka et al., 1999).

Mammalian studies of TD pressure have been equally divergent. Early manometric investigations on anesthetized dogs reported peak pressures at the end of expiration, with declines during inspiration. Labored breathing (via administration of large quantities of ether) induced pressure elevation that dissipated with respiratory cessation (Lee, 1924). Later studies confirmed alternating positive and negative pressure waves seen with respiratory movements, and a pressure wave corresponding to cardiac systole, in addition to visible oscillations independent of either, were present at the level of the cisterna chyli. Synchronicity with inspiration or expiration was not stated (Conforti et al., 1955). Hall et al. replicated similar findings, as well as reporting that the amplitude of pressure waves depended on the rate and depth of inspiration (Hall et al., 1965). Pflug and Calnan observed expiratory pressure peaks and fine 1–2 mmHg pressure waves in time with cardiac pulsation, but no intrinsic contractile activity (Pflug & Calnan, 1968). These findings were later replicated by the same authors, where respiration‐related pressure changes (5 mmHg) exceeded arterial ones (1 mmHg), and an end‐expiratory rise in pressure was attributed to the closure of the LVJ valve (Calnan et al., 1970). Browse et al. later demonstrated that pressure within the TD varied by vertebral level and respiratory phase, with negative pressure on inspiration, and that arterial pulsation transmitted pressure waves to adjacent duct segments. Conscious dogs had lower baseline TD pressures than mechanically ventilated ones in this study, although levels gradually increased after a 100–200 mL drink of water. No evidence of spontaneous TD contractions was found in either group (Browse et al., 1971).

Collectively, these animal studies underscore the complexity of the TD's physiology (Table 2). While respiration and cardiac pulsation are well‐documented factors modulating TD lymph flow and pressure, there is mixed evidence for a significant contribution from intrinsic contractility of the duct. Differences in species, anesthetic protocols, ventilation parameters, and measurement techniques all contribute to the divergent results reported.

**TABLE 2:** The summary of impact of respiration and circulation on TD flow and pressure in animals as reported in the literature (Browse et al., 1971, 1974; Calnan et al., 1970; Conforti et al., 1955; Fazzini et al., 1964b; Hall et al., 1965; Hatta, 1952; Iizuka et al., 1999; Kitamura, 1967; Lee, 1924; Nelson et al., 1969, 1970; Nusbaum et al., 1964; Onizuka et al., 1997; Pflug & Calnan, 1968; Pilon & Bittar, 1973; Schad et al., 1978).

Author	Subject	Sample size	Method	Flow or pressure synchronous with respiration	Flow or pressure synchronous with heart rate	Intrinsic contractions of TD observed
Lee (1924)	Dogs, anesthetized with ether vapor via intratracheal insufflation method, and in which all lymph trunks except the TD, as well as internal jugular, subclavian, external jugular and innominate (brachiocephalic) veins had been ligated	9	Cannulation of external jugular vein with T‐shaped glass tube, to the other arm of which a rubber tube was attached to collect lymph (so that contents of TD were directed solely into the tube). Stem of T‐tube connected to manometer with a writing arm that recorded changes in lymph‐pressure	Yes. Peak of lymph‐pressure reached at the end of expiration	Pressure changes associated with pulse rate “visible to the naked eye”, but too small to be recorded with the manometer used	N/A
Hatta (1952)	Dogs, further details unspecified	Unspecified	Cannulation of the cisterna chyli and venous end of the TD, and duct perfused with Locke's solution with constant pressure of 10–15 cmH_2_O. Flow measured by recording the number of drops per unit time of perfusate	Perfusate from the TD flowed out in sync with expiration and stopped with inspiration. Stopping or slowing respiration (via vagal stimulation) caused stopping or decrease of flow	N/A	Suggested, as flow from the TD increased and decreased rhythmically even following the death of the animals
Conforti et al. (1955)	Dogs anesthetized with ether via endotracheal intubation	Unspecified	Cannulation of the TD with a polythene tube, and pressure curves recorded with an electro‐manometer	Each respiratory movement produced pressure waves that were alternatively positive and negative (measurements not given). Synchrony with inspiration or expiration not stated	A pressure oscillation was seen corresponding to every cardiac systole (measurement not given)	Slight pressure oscillations independent from respiratory movements and the cardiac rhythm were observed
Nusbaum et al. (1964)	Anesthetized, spontaneously breathing mongrel dogs	10	Fluoroscopic lymphangiography using chlorophylised Ethiodol dye	No	Visually observed, but not confirmed on monitoring	Flow resembling propulsion by TD peristalsis observed but definite TD contractions could not be confirmed
Fazzini et al. (1964a) [Note: inaccuracies possible due to mistranslation from Italian to English]	Dogs, further details unspecified	9	TD was cannulated and lymph collected (collection method not stated in paper). Lymph flow rates were recorded for 1 h both before and after bilateral phrenicotomies performed	There was no significant difference in lymph flow rate before and after diaphragmatic paralysis via bilateral phrenicotomy. (15.05 ± 0.42 mL/h. vs. 16.7 ± 0.69 mL/h)	N/A	N/A
Hall et al. (1965)	Conscious sheep, in which chronic lymphatic fistulae were established in numerous lymph trunks and ducts	100+	Cannulation of the TD at both cranial and caudal end, with lymph flow rates measured by collecting lymph in graduated vessels and pressure transmitted via T‐tube to an inductive transducer then recorded by galvanometer recorder. Respiration rate counted manually. Other lymphatic vessels were also investigated in this study	Pressure fluctuations were present at the cranial end of the TD that were said to be due to respiration, however pressure tracing with respiration for TD not given, nor was synchronicity with inspiration or expiration commented on	N/A	Pressure fluctuations present that were reportedly due to intrinsic duct contractility and were slower and of lesser amplitude than those from respiration, and of those from the intrinsic activity of other lymphatic vessels. However, pressure tracing with simultaneous respiration counts not given
Kitamura (1967) [Note: inaccuracies possible due to mistranslations from Japanese to English]	Dogs–further details unobtainable	37	Cannulation of the TD with a vinyl tube in 7 dogs at LVJ and just above diaphragm, and in 30 more dogs, TD and right lymphatic duct cannulated at both venous angles	Concluded that TD lymph flow is accelerated by the fall in cervical venous pressure in the inspiratory phase and contraction of the diaphragm in the expiratory phase. Cessation of diaphragm movement was noted to significantly reduce TD lymph flow	N/A	N/A
Pflug & Calnan (1968)	Dogs under “light anesthesia”, further details unspecified	18	Cannulation of the TD and jugular vein with polyethylene tubes, which were connected to strain‐gauge transducers and pressures recorded by pen tracing on paper tape. Synchronous side‐pressures of TD and jugular vein were recorded in some. The TD was also dissected and examined under microscope in 20 dogs and 7 human cadavers	At the beginning of expiration, the pressure in TD rose to a maximum, and appeared to fall during inspiration. TD pressure rose on Valsalva maneuver	Fine TD pressure waves of 1–2 mmHg were seen that were synchronous with heart rate–unspecified if on systole or diastole	No
Nelson et al. (1969)	Anesthetized dogs, given either propranolol, phentolamine or isoproterenol, or made hypotensive by bleeding. Control group was given no drugs and not made hypotensive	42	Cannulation of the TD, and lymph flow measured with a drop counter. Vitals recorded continuously. Femoral vein cannulated for administration of drugs	N/A	Pulse rate did not correlate with TD lymph flow	N/A
Nelson et al. (1970)	Anesthetized dogs, given various alpha‐ and beta‐adrenergic blocking or activating drugs, or made hypotensive by bleeding or via endotoxins. Control group was given no drugs and not made hypotensive	90	Cannulation of the TD, and lymph flow measured with a drop counter. Vitals recorded continuously. Femoral vein cannulated for administration of drugs	N/A	Pulse rate did not correlate with TD lymph flow	N/A
Calnan et al. (1970)	Adult greyhounds under general anesthesia	23	Cannulation of the TD with polyethylene tubing, connected to strain‐gauge transducers with pressure recorded by pen on paper tape. Cannulas were also united by T‐tubes, so lymph flow could continue while side pressures recorded. Unclear how/if respiration and heart rate measured	There was a TD pressure wave synchronous with respiration (unclear which part of the cycle), with an amplitude of about 5 mmHg	A smaller, more rapid TD pressure wave was present synchronous with the pulse rate, with an amplitude of less than 1 mmHg	N/A
Browse et al. (1971)	Conscious dogs and anesthetized dogs (both spontaneously breathing and artificially ventilated with positive pressure ventilation)	20	Cannulation of the TD at the T2 and T5 or T10 vertebral levels. Cannulas connected to transducers to measure pressure. Arterial blood pressure and respiration also measured. Some dogs were artificially ventilated. The aorta was obstructed with a balloon catheter in some experiments	A small, negative TD pressure wave occurred during inspiration in the spontaneously breathing anesthetized dog. The direction of this was reversed in the artificially ventilated dog, in which the pressure wave was positive on inspiration. This was observed in the TD at both T5 and T10 vertebral levels	A small, regular positive TD pressure wave was seen which was synchronous with peaks in aortic blood pressure. Obstructing the aorta produced changes in these pressure waves	No pressure waves present which could be attributed to intrinsic spontaneous contractions of the duct
Pilon & Bittar (1973)	Anesthetized dogs on PEEP ventilation, respiratory rate 24 min^−1^	8	Cannulation of the TD at both ends with a polyethylene tube, free ends exteriorised separately and joined by a bubble flowmeter, which was used to measure flow rate before returning lymph to the circulation	Starting PEEP caused an immediate significant reduction in TD lymph flow, which then gradually increased over the next half hour	N/A	N/A
Browse et al. (1974)	Conscious dogs and anesthetized dogs (both spontaneously breathing and artificially ventilated with positive pressure ventilation)	7	Ultrafluid lipiodol (radio‐opaque oil) was injected into the lymphatics via a catheter, and the velocity of lymph flow was measured by timing the oil droplets traveling along 10 cm sections of duct. Cannulas were inserted into the thoracic aorta and great veins, to measure pressures (via transducer)	Flow was intermittent, with the main cephalad movement occurring during and shortly after inspiration, with the fastest movement around the thoraco‐lumbar region. The rate of flow was faster overall in the ventilated dog, and greater yet in the conscious dogs	There were smaller movements of forward and backwards lymph flow which corresponded to aortic pulsations. The forward movement was approximately the same as the backwards movement, so it did not contribute to overall forward flow. This phenomenon was only investigated in the anesthetized dogs	N/A
Schad & Brechtelsbauer (1978)	Anesthetized, artificially ventilated dogs	12	Cannulation of the TD and aorta (for BP monitoring). TD lymph flow was measured via graded tube. The 5th intercostal space was opened bilaterally 10–15 cm to reversibly reduce respiratory intrathoracic pressure changes: either kept open with a retractor or closed by clamps. Other possible lymph driving forces (saline infusion, passive hind limb movement, hyperventilation) were also investigated	Hyperventilation led to an increase in TD lymph flow–however, synchronicity of flow with the phases of the respiratory cycle was not investigated. Opening the thorax reduced TD flow	N/A	N/A
Onizuka et al. (1997)	Anesthetized sheep on PEEP ventilation, and conscious sheep	11	TD lymph flow was measured with an ultrasound transit‐time flow probe connected to a flowmeter, introduced via thoracotomy. A catheter was placed in the right external jugular vein, by which outflow venous pressure of the TD was measured	Lymph flow showed no relation to airway pressure fluctuations, in both anesthetized and conscious sheep. Stopping ventilation produced no change in lymph flow pattern	Lymph flow showed no relation to arterial blood pulsation in anesthetized and conscious sheep	Lymph flow in the TD was pulsatile and independent of airway and arterial pressure
Iizuka et al. (1999)	Anesthetized sheep on positive pressure ventilation	10	TD lymph flow was measured with an ultrasound transit‐time flowmeter positioned on the TD (via right thoracotomy) and digitally recorded. The right external jugular vein was also cannulated to measure the venous outflow pressure of the TD using a transducer	The TD flow wave form included a high frequency component (24 min^−1^), which was said to represent respiratory movement. Synchronicity with inspiration or expiration not investigated	The TD flow wave form included a 180 min^−1^ fluctuation, which was attributed to cardiac pulsation	A low frequency component of the TD flow wave form (~0.1 Hz) was present and thought to be from intrinsic TD pulsation

Abbreviations: ICS, intercostal space; PEEP, positive end‐expiratory pressure; TD, thoracic duct.

## 4 DISCUSSION

There is currently no consensus on the basic fluid dynamics, in terms of flow and pressure, in the human TD. Direct evidence for both intrinsic activity and the contribution and nature of extrinsic propulsive forces such as transmitted arterial pulsations and the respiratory cycle is limited and widely discordant in the literature. The mechanism(s) by which lymph travels through the TD and into the venous system therefore remain surprisingly unclear. Recently much attention has been directed towards developing comprehensive computational models of lymph flow, namely by elucidating the dynamics of lymphatic valves and the rhythmic pumping action of lymphangions, which are the functional units of lymphatic vessels that lie between two semilunar valves (Jayathunga et al., 2024; Jayathungage Don et al., 2024). While these efforts have certainly added insight and increased the sophistication of our understanding of how the lymphatic network moves fluid at large, these models have not focused on the TD itself. There are many reasons that the TD is unique from its tributaries, including its diameter, its terminal communication with the venous system, its scant muscular coat and irregular valve distribution, its location in the thorax and relations to adjacent large arteries (El Zawahry et al., 1983; Jacobsson, 1972). For all these reasons, the TD needs to be studied directly, and inferences not made from other lymphatic vessels.

The significant lack of consensus in study findings may be due to varying study methods, differences between (upright) human and (horizontal) non‐human mammal subjects, healthy versus diseased states, spontaneously respiring versus mechanically ventilated subjects, and observational error due to disparate techniques for measuring lymph flow and pressure. Anesthesia and mechanical ventilation alter intrathoracic pressure dynamics and lymphatic smooth muscle contractility, meaning that findings derived from these subjects may not reflect physiological lymphatic behavior in awake, spontaneously breathing conditions (Bachmann et al., 2019; Dull et al., 2024; McHale & Thornbury, 1989; Schad & Brechtelsbauer, 1978). Other factors potentially contributing to or affecting the movement of TD lymph have also been reported, including central venous pressure (via impact of the pressure gradient between TD and central veins affecting flow rate), gravity and positioning, traumatic injury, exercise and passive movement, chemical agents and more (Demchenko & Vovk, 1991; Desai et al., 2010; Dolley & Wiese, 1929; Downey et al., 2008; Elkins et al., 1953; Entrup et al., 1966; Foldi & Zoltan, 1966; Grindlay et al., 1948; Haider et al., 1987; Hatta, 1952; Kinnaert, 1973; Knott et al., 2005; Nelson et al., 1969, 1970; Pilon & Bittar, 1973; Schad et al., 1978; Schad & Brechtelsbauer, 1977). Furthermore, as lymphatic contractile function is exquisitely sensitive to flow and preload (which are determined by upstream lymphatic flow), factors such as hydration status, capillary permeability and metabolic state (fed or fasted) of the subject are likely to influence intrinsic contractility of the TD.

Reports of the volume of lymph drained from the human TD via a catheter vary from 200 mL to 8 L per day (Russell et al., 2022). One study of cirrhotic patients reported a range of 3–7 mL/min, with cannulation of the TD and collection of lymph over a 10‐minute period (Dumont et al., 1960). Several attempts have been made to investigate how this flow might be related to the respiratory cycle and intrathoracic pressure changes. Inspiration drops intrathoracic pressure and increases intra‐abdominal pressure (Carroll, 2007; Préau et al., 2012). The effect of these changes on the movement of lymph in the TD is difficult to ascertain; there are contradictory results regarding which part of the breathing cycle produces the greatest flow. For example, a study suggested that TD pressure decreases during inspiration, potentially favoring a cephalad movement of lymph, and that the decrease in abdominal pressure during expiration may allow the cisterna chyli (CC) to fill, while the increase in intrathoracic pressure may allow lymph to overcome central venous pressure and enter the circulation (Kelly et al., 2022). Another found that the smallest mean diameter of the terminal TD was seen at full expiration, although flow itself was not visualized (Hinton et al., 2022).

The study method of choice influences which portion of the TD is measured, whether the average rate of flow across its entire TD length is obtained, or the progression of lymph across a particular segment, or only its transit through the LVJ. At a specific time point, different flow velocities could exist at different parts of the TD, with potentially varying physiology. A similar quandary exists for pressure studies and there is disagreement regarding the continuity of flow, as some studies found it to be free‐flowing while others reported it was intermittent or both, depending on the portion of duct studied (Bradham & Takaro, 1968; Browse et al., 1974; Calnan et al., 1970; El Zawahry et al., 1983; Nusbaum et al., 1964; Pflug & Calnan, 1968; Riemenschneider & Shields, 1981). The presence of valves in the TD and at the LVJ could also affect flow and pressure. At the LVJ, bicuspid semilunar valves may be present, with a theorized function of preventing blood from entering the lymphatic system (O'Hagan et al., 2020). Pflug and Calnan hypothesized that the valves at the LVJ close during inspiration and open during expiration to provide an open channel for lymph to enter the blood circulation (Pflug & Calnan, 1968). The issue here is that there is no consistency to the presence or configuration of valves at the terminal LVJ.

The impact of cardiac pulsation is more consistently described in the literature, with most authors noting a change in flow or pressure corresponding to pulse rate, postulating that the aorta or left atrium has a percussive effect on the adjacent thoracic duct (Browse et al., 1971; El Zawahry et al., 1983; Kinnaert, 1973; Nusbaum et al., 1964; Pflug & Calnan, 1968). The exact physiology remains unclear. There is minimal discussion in the literature of the TD's relation to fascial layers and its exact positioning with respect to the great vessels; however, a recent study by Hirano et al. aimed to clarify this. The study consisted of human cadaveric dissection, observation of anatomical sections from an online open resource, and 3D reconstruction of these sections. The TD was observed to run closely adjacent to large arteries, namely the aorta, common carotid artery, and subclavian artery for most of its course, and entered the mediastinum sandwiched in between the aorta and the pleura. However, the aorta was located anteriorly away from the TD around the T5 vertebral level, and from that level up until it deviated to the left and ascended along the left common carotid artery to terminate at the LVJ, the TD was not found to be closely accompanying the large arteries. The study also investigated the TD length and course during inspiration and expiration via measurement on CT images but relied on landmarks based on surrounding arteries as the TD is difficult to visualize on plain CT (Hirano et al., 2025). Thus, it remains unclear if movement of thoracic organs (lung expansion, diaphragm contraction, cardiac and aortic pulsation) has an influence on the flow of the lymphatic vessel via direct compression, intra‐cavity pressure changes, or a combination of these two mechanisms and/or others.

In both human and non‐human mammalian studies, there are mixed results on the presence, or lack of, intrinsic contractile activity of the TD. This is most likely due to experimental technique; some methods may not be sensitive enough to detect these contractions, or the contractile activity of the TD may be altered by more invasive study methods such as cannulation. Cannulating the TD could also distort the overall anatomy and pressure relationships; hence advanced non‐invasive imaging techniques will be necessary for further advances. These modern advancements in lymphatic imaging will allow for more direct emphasis to be placed on living, spontaneously breathing, healthy human subjects, eliminating the need for other mammals which have differences in the mechanisms of lymph flow and pressure. In the sheep or canine, the TD typically remains horizontal, eliminating the effect of gravity compared to upright humans. In dogs, gravity was found to reduce TD flow rate and the upward progression towards the LVJ, and orienting the TD so it is upright decreased pressure in its upper segment (Entrup et al., 1966). Additionally, the state of consciousness of the subject may change the physiology of the TD, either from the effect of mechanical ventilation alone, or from decreased skeletal muscle contraction and sympathetic stimulation. The augmentation of mechanical ventilation on lymph return has been inconsistently reported in humans and dogs. A significant rise in TD and central venous pressure occurs during positive pressure ventilation, compared to a decrease during spontaneous respiration (Kelly et al., 2022). PEEP ventilation causes an immediate significant reduction of lymph flow in dogs, but also increases lymph formation, so that flow increases gradually after PEEP is commenced (Haider et al., 1987; Pilon & Bittar, 1973). It has also been suggested that the effect of respiration on TD flow is less in conscious compared with anesthetized non‐human mammals (Schad & Brechtelsbauer, 1978). This effect may be compounded by the effect of the anesthetic agents themselves, as it has been shown that certain anesthetic agents have an inhibitory effect on lymphatic contractility in a dose‐dependent manner, sometimes abolishing the contractions completely (Bachmann et al., 2019; Dull et al., 2024; McHale & Thornbury, 1989). Furthermore, TD lymph flow has been found to decrease in spontaneously breathing, anesthetized dogs, when compared to conscious dogs (Schad & Brechtelsbauer, 1977). As anesthesia and mechanical ventilation introduce significant alterations in intrathoracic pressure and lymphatic contractility, cautious interpretation of these studies is necessary.

## 5 CONCLUSION

Overall, the existing evidence on TD physiology remains limited, inconsistent, and highly heterogeneous. The reviewed studies span several decades, differ greatly in methods, species, ventilation states, and measurement approaches, and often lack sufficient detail to allow meaningful comparison. Importantly, fewer than 1% of screened studies met the inclusion criteria, highlighting the limitations in data on this topic. Consequently, this review should be interpreted as a prospective on the knowledge gap, rather than a definitive physiological synthesis.

Further research requires a more rigorous and technologically advanced approach. Modern imaging modalities, such as dynamic MR lymphangiography, near infrared fluorescence imaging, and high frame rate ultrasound, should be used to directly visualize TD flow in real time. Studies should report key physiological variables that influence lymphatic dynamics, including ventilation mode (spontaneous vs. mechanical), intrathoracic pressure, posture, hydration status, central venous pressure, and anesthetic state. Importantly, future work should prioritize simultaneous measurement of TD pressure, flow, and respiratory rate and circulatory rhythms, ideally in awake human subjects. Clear, standardized definitions of flow, pressure, and valve behavior are also necessary to allow comparison across studies.

These steps will enable accurate, reproducible, and clinically relevant characterization of TD physiology and establish the foundations needed for translational applications in lymphatic and cardiovascular medicine.

## AUTHOR CONTRIBUTIONS

S.M.: investigation, formal analysis, writing—original draft; L.A.O: investigation, formal analysis, validation, writing—review and editing; J.A.W, A.R.J.P., P.S.R., A.R.C: writing—review & editing; S.A.M.: conceptualization, methodology, supervision, writing—review and editing.

## FUNDING INFORMATION

The authors have nothing to report.

## ETHICS STATEMENT

This is a review paper and it is not relevant.

## Supporting information


**Data S1.** Supporting Information.
